# Comprehensive registry of esophageal cancer in Japan, 2013

**DOI:** 10.1007/s10388-020-00785-y

**Published:** 2020-10-13

**Authors:** Masayuki Watanabe, Yuji Tachimori, Tsuneo Oyama, Yasushi Toh, Hisahiro Matsubara, Masaki Ueno, Koji Kono, Takashi Uno, Ryu Ishihara, Kei Muro, Hodaka Numasaki, Koji Tanaka, Soji Ozawa, Kentaro Murakami, Shiyori Usune, Arata Takahashi, Hiroaki Miyata, The Registration Committee for Esophageal Cancer of the Japan Esophageal Society

**Affiliations:** 1 Department of Gastroenterological Surgery, Cancer Institute Hospital of Japanese Foundation for Cancer Research, Tokyo, Japan; 2 Cancer Care Center, Kawasaki Saiwai Hospital, Kawasaki, Kanagawa Japan; 3 Department of Endoscopy, Saku Central Hospital Advanced Care Center, Saku Nagano, Japan; 4 Department of Gastroenterological Surgery, National Hospital Organization Kyushu Cancer Center, Fukuoka, Japan; 5 Department of Frontier Surgery, Graduate School of Medicine, Chiba University, Chiba, Japan; 6 Department of Gastroenterological Surgery, Toranomon Hospital, Tokyo, Japan; 7 Department of Gastrointestinal Tract Surgery, Fukushima Medical University School of Medicine, Fukushima, Japan; 8 Department of Diagnostic Radiology and Radiation Oncology, Graduate School of Medicine, Chiba University, Chiba, Japan; 9 Department of Gastrointestinal Oncology, Osaka International Cancer Institute, Osaka, Japan; 10 Department of Clinical Oncology, Aichi Cancer Center Hospital, Nagoya, Japan; 11 Department of Medical Physics and Engineering, Graduate School of Medicine, Osaka University, Suita, Osaka, Japan; 12 Department Gastroenterological Surgery, Graduate School of Medicine, Osaka University, Osaka, Japan; 13 Department of Gastroenterological Surgery, Tokai University School of Medicine, Isehara, Kanagawa Japan; 14 Department of Healthcare Quality Assessment, Graduate School of Medicine, The University of Tokyo, Tokyo, Japan

**Keywords:** Esophageal cancer, Esophagectomy, Endoscopic resection, Chemotherapy, Chemoradiotherapy

## Abstract

**Background:**

Esophageal cancer is the eighth most common cause of cancer mortality in Japan. More than 11,000 people had died from esophageal cancer in 2018. The Japan Esophageal Society has collected the data on patients' characteristics, performed treatment, and outcomes annually.

**Methods:**

We analyzed the data of patients who had first visited the participating hospitals in 2013. In 2019, the data collection method was changed from an electronic submission to a web-based data collection using the National Clinical Database (NCD). Japanese Classification of Esophageal Cancer 10th by the Japan Esophageal Society (JES) and UICC TNM Classification 7th were used for cancer staging

**Results:**

A total of 8019 cases were registered from 334 institutions in Japan. Squamous cell carcinoma and adenocarcinoma accounted for 87.8% and 6.3%, respectively. The 5-year survival rates of patients treated using endoscopic resection, concurrent chemoradiotherapy, radiotherapy alone, or esophagectomy were 88.3%, 32.4%, 24.4%, and 59.3%, respectively. Esophagectomy was performed in 4910 cases. The operative and the hospital mortality rates were 0.77% and 1.98%, respectively. The survival curves showed a good discriminatory ability both in the clinical and pathologic stages by the JES system. The 5-year survival rate of patients with pStage IV in the UICC classification that included patients with supraclavicular node metastasis was better than that of patients with pStage IVb in JES classification.

**Conclusion:**

We hope this report contributes to improving all aspects of the diagnosis and treatment of esophageal cancer in Japan.

## Preface 2013

We deeply appreciate the great contributions of many physicians in the registry of esophageal cancer cases. The Comprehensive Registry of Esophageal Cancer in Japan, 2013, was published here. In 2019, the data collection method was changed from an electronic submission to a web-based data collection using the National Clinical Database (NCD). Personal information was replaced with individual management code inside each institute, and the NCD collected only anonymized information. The registry complies with the Act for the Protection of Personal Information.

We briefly summarized the Comprehensive Registry of Esophageal Cancer in Japan, 2013. Japanese Classification of Esophageal Cancer 10th by the Japan Esophageal Society (JES) [1] and UICC TNM Classification 7th [2] were used for cancer staging according to the subjected year. A total of 8019 cases were registered from 334 institutions in Japan. Tumor locations were cervical: 4.8%, upper thoracic: 12.1%, middle thoracic: 46.5%, lower thoracic: 28.2% and EG junction: 7.9%. Superficial carcinomas (Tis, T1a, T1b) were 38.6%. As for the histologic type of biopsy specimens, squamous cell carcinoma and adenocarcinoma accounted for 87.8% and 6.3%, respectively. Regarding clinical results, the 5-year survival rates of patients treated using endoscopic resection, concurrent chemoradiotherapy, radiotherapy alone, or esophagectomy were 88.3%, 32.4%, 24.4%, and 59.3%, respectively. The endoscopic submucosal dissection accounted for 91.6% of endoscopic resection. Esophagectomy was performed in 4910 cases. Concerning the approach used for esophagectomy, 43.0% of the cases were treated thoracoscopically. The operative mortality (within 30 days after surgery) was 0.77%, and the hospital mortality was 1.98%. The Kaplan–Meier survival curves diverged according to the N-grade both in the JES and the UICC classifications. The survival curves showed a good discriminatory ability both in the clinical and pathologic stages by the JES system. However, the survival of cStage IIB was better than those of IB and IIA, while the survival curves were almost identical between cStage IIIc and IV in the UICC system. Also, the survival curve of pStage IIA merged with that of IIB, and the survival of pStage IV was better than that of IIIC. The 5-year survival rate of patients with pStage IV in the UICC classification that included patients with supraclavicular node metastasis was better than that of patients with pStage IVb in JES classification.

We hope that this Comprehensive Registry of Esophageal Cancer in Japan for 2013 will help to improve all aspects of the diagnosis and treatment of esophageal cancer in Japan.

## Contents

I. **Clinical factors of esophageal cancer patients treated in 2013**
  1. **Institution-registered cases in 2013**
  2. **Patient background**
    **Table** **1****Age and gender**
    **Table** **2****Primary treatment**
    **Table** **3****Tumor location**
    **Table** **4****Histologic types of biopsy specimens**
    **Table** **5****Depth of tumor invasion, cT (UICC TNM 7th)**
    **Table** **6****Lymph node metastasis, cN (UICC TNM 7th)**
    **Table** **7****Distant metastasis, cM (UICC TNM 7th)**
    **Table** **8****Clinical stage (UICC TNM 7th)**
II. **Results of endoscopically treated patients in 2013**
  **Table** **9****Details of endoscopic treatment for curative intent**
  **Table** **10****Complications of EMR/ESD**
  **Table** **11****Pathological depth of tumor invasion of EMR/ESD specimens**
  **Figure** **1****Survival of patients treated with EMR/ESD**
  **Figure** **2****Survival of patients treated with EMR/ESD according to the pathological depth of tumor invasion, pT (JES 10th)**
  **Figure** **3****Survival of patients treated with EMR/ESD according to the lymphatic and venous invasion**
III. **Results in patients treated with chemotherapy and/or radiotherapy in 2013**
  **Table** **12****Dose of irradiation (non-surgically treated cases)**
  **Table** **13****Dose of irradiation (surgically treated cases)**
  **Figure** **4****Survival of patients treated with chemotherapy and/or radiotherapy**
  **Figure** **5****Survival of patients treated with definitive chemoradiotherapy according to clinical stage (UICC TNM 7th)**
  **Figure** **6****Survival of patients underwent radiotherapy alone according to clinical stage (UICC TNM 7th)**
IV. **Results in patients who underwent esophagectomy in 2013**
  **Table** **14****Treatment modalities of esophagectomy**
  **Table** **15****Tumor location**
  **Table** **16****Approaches to tumor resection**
  **Table** **17****Video-assisted surgery**
  **Table** **18****Fields of lymph node dissection according to the location of the tumor**
  **Table** **19****Reconstruction route**
  **Table** **20****Organs used for reconstruction**
  **Table** **21****Histological classification**
  **Table** **22****Depth of tumor invasion, pT (JES 10th)**
  **Table** **23****Pathological grading of lymph node metastasis, pN (JES 10th)**
  **Table** **24****Pathological findings of lymph node metastasis, pN (UICC TNM 7th)**
  **Table** **25****Pathological findings of distant organ metastasis, pM (JES 10th)**
  **Table** **26****Residual tumor**
  **Table** **27****Causes of death**
  **Figure** **7****Survival of patients who underwent esophagectomy**
  **Figure** **8****Survival of patients who underwent esophagectomy according to clinical stage (JES 10th)**
  **Figure** **9****Survival of patients who underwent esophagectomy according to clinical stage (UICC TNM 7th)**
  **Figure** **10****Survival of patients who underwent esophagectomy according to the depth of tumor invasion, pT (JES 10th)**
  **Figure** **11****Survival of patients who underwent esophagectomy according to lymph node metastasis (JES 10th)**
  **Figure** **12****Survival of patients who underwent esophagectomy according to lymph node metastasis (UICC TNM 7th)**
  **Figure** **13****Survival of patients who underwent esophagectomy according to pathological stage (JES 10th)**
  **Figure** **14****Survival of patients who underwent esophagectomy according to pathological stage (UICC TNM 7th)**
  **Figure** **15****Survival of patients who underwent esophagectomy according to residual tumor (R)**

## I. Clinical features of esophageal cancer patients treated in 2013

Institution-registered cases in 2013.

**Table:** 

Institutions
Ageo Central General Hospital
Aichi Cancer Center
Aichi Medical University Hospital
Aizawa Hospital
Akita University Hospital
Aomori Prefectural Central Hospital
Arao Municipal Hospital
Asahikawa Medical University Hospital
Cancer Institute Hospital of JFCR
Chiba Cancer Center
Chiba University Hospital
Chibaken Saiseikai Narashino Hospital
Chiba-Nishi General Hospital
Chigasaki Municipal Hospital
Chugoku Rosai Hospital
Dokkyo Medical University Hospital
Ehime Prefectural Central Hospital
Eijyu General Hospital
Fuchinobe General Hospital
Fuchu Hospital
Fujinomiya City General Hospital
Fujioka General Hospital
Fujisaki Hospital
Fujita Health University Hospital
Fukaya Red Cross Hospital
Fukui Prefectural Hospital
Fukui University Hospital
Fukui-ken Saiseikai Hospital
Fukuoka City Hospital
Fukuoka Shin Mizumaki Hospital
Fukuoka University Chikushi Hospital
Fukuoka University Hospital
Fukuoka Wajiro Hospital
Fukushima Medical University Hospital
Fukuyama City Hospital
Gifu Prefectural General Center
Gifu University Hospital
Gunma Prefectural Cancer Center
Gunma Saiseikai Maebashi Hospital
Gunma University Hospital
Hachinohe City Hospital
Hakodate City Hospital
Hakodate Goryokaku Hospital
Hakodate National Hospital
Hamamatsu University Hospital
Heartlife Hospital
Higashiosaka City Medical Center
Hiraka General Hospital
Hiratsuka City Hospital
Hiratsuka Kyosai Hospital
Hirosaki University Hospital
Hiroshima City Asa Hospital
Hiroshima City Hospital
Hiroshima Red Cross Hospital & Atomic-bomb Survivors Hospital
Hiroshima University Hospital
Hitachi General Hospital
Hofu Institute of Gastroenterology
Hokkaido University Hospital
Hospital of the University of Occupational and Environmental Health, Japan
Hyogo Cancer Center
Hyogo Prefectural Amagasaki General Medical Center
Ibaraki Prefectural Central Hospital
Iizuka Hospital
International Goodwill Hospital
International University of Health and Welfare Atami Hospital
International University of Health and Welfare Hospital
International University of Health and Welfare Ichikawa Hospital
International University of Health and Welfare Mita Hospital
Isehara Kyodo Hospital
Ishikawa Prefectural Central Hospital
Itami City Hospital
Iwata City Hospital
Iwate Medical University Hospital
Iwate Prefectural Central Hospital
Iwate Prefectural Chubu Hospital
JA Hiroshima General Hospital
JA Kouseiren Enshu Hospital
JA Onomichi General Hospital
Japanese Red Cross Ashikaga Hospital
Japanese Red Cross Fukuoka Hospital
Japanese Red Cross Ishinomaki Hospital
Japanese Red Cross Kitami Hospital
Japanese Red Cross Kyoto Daiichi Hospital
Japanese Red Cross Maebashi Hospital
Japanese Red Cross Medical Center
Japanese Red Cross Musashino Hospital
Japanese Red Cross Nagasaki Genbaku Hospital
Japanese Red Cross Nagoya Daiichi Hospital
Japanese Red Cross Saitama Hospital
Japanese Red Cross Society Nagano Hospital
Japanese Red Cross Tottori Hospital
Japanese Red Cross Wakayama Medical Center
JCHO Gunma Chuo Hospital
JCHO Kyushu Hospital
JCHO Miyazaki Konan Hospital
JCHO Osaka Hospital
JCHO Saitama Medical Center
JCHO Tokuyama Central Hospital
JCHO Yokohama Chuo Hospital
Jichi Medical University Hospital
Jichi Medical University Saitama Medical Center
Juntendo University Hospital
Juntendo University Shizuoka Hospital
Juntendo University Urayasu Hospital
Junwakai Memorial Hospital
Kagawa Prefectural Central Hospital
Kagawa Rosai Hospital
Kagawa University Hospital
Kagoshima University Hospital
Kaizuka City Hospital
Kakogawa Central City hospital
Kanagawa Cancer Center
Kanazawa Medical University Hospital
Kanazawa University Hospital
Kansai Denryoku Hospital
Kansai Medical University Hospital
Kansai Medical University Medical Center
Kansai Rosai Hospital
Kanto Central Hospital
Kashiwa Kousei General Hospital
Kasugai Municipal Hospital
Kawasaki Hospital
Kawasaki Medical School Hospital
Kawasaki Medical School Kawasaki Hospital
Kawasaki Municipal Hospital
Kawasaki Municipal Ida Hospital
Kawasaki Saiwai Hospital
Keio University Hospital
Keiyukai Sapporo Hospital
Kindai University Hospital
Kindai University Nara Hospital
Kinki Central Hospital
Kiryu Kousei General Hospital
Kitaakita Municipal Hospital
Kitaharima Medical Center
Kitakyushu General Hospital
Kitakyushu Municipal Medical Center
Kitano Hospital
Kitasato University Hospital
Kobe City Medical Center General Hospital
Kobe University Hospital
Kochi Health Science Center
Kochi University Hospital
Kokura Memorial Hospital
Kouseiren Takaoka Hospital
Kumagai General Hospital
Kumamoto University Hospital
Kummoto Regional Medical Center
Kurashiki Central Hospital
Kurume University Hospital
Kyorin University Hospital
Kyoto University Hospital
Kyoto-Katsura Hospital
Kyushu Central Hospital
Kyushu University Hospital
Machida Municipal hospital
Matsudo City General Hospital
Matsushita Memorial Hospital
Matsuyama Red Cross Hospital
Mie University Hospital
Minamiosaka Hospital
Minoh City Hospital
Mito Red Cross Hospital
Mitsui Memorial Hospital
Miyazaki University Hospital
Mizushima Kyudo Hospital
Moriguchi Keijinkai Hospital
Murakami General Hospital
Nagahama City Hospital
Nagahama Red Cross Hospital
Nagano Municipal Hospital
Nagaoka Chuo General Hospital
Nagasaki University Hospital
Nagoya City University Hospital
Nagoya City West Medical Center
Nagoya University Hospital
Nanpuh Hospital
Nara City Hospital
Nara Medical University Hospital
Nasu Red Cross Hospital
National Cancer Center Hospital
National Cancer Center Hospital East
National Center for Global Health and Medicine
National Defence Medical College Hospital
New Tokyo Hospital
NHO Beppu Medical Center
NHO Chiba Medical Center
NHO Fukuoka-Higashi Medical Center
NHO Iwakuni Clinincal Center
NHO Kanmon Medical Center
NHO Kure Medical Center
NHO Kyoto Medical Center
NHO Kyushu Cancer Center
NHO Matsumoto Medical Center
NHO Mito Medical Center
NHO Miyakonojo Medical Center
NHO Nagasaki Medical Center
NHO Nagoya Medical Center
NHO Okayama Medical Center
NHO Osaka Medical Center
NHO Saitama Hospital
NHO Sendai Medical Center
NHO Shikoku Cancer Center
NHO Tokyo Medical Center
NHO Yokohama Medical Center
Nihonkai General Hospital
Niigata Cancer Center Hospital
Niigata City General Hospital
Niigata Prefectural Central Hospital
Niigata Prefectural Shibata Hospital
Niigata University Medical & Detal Hospital
Nikko Memorial Hospital
Nippon Medical School Chiba Hokusou Hospital
Nippon Medical School Hospital
Nippon Medical School Musashi Kosugi Hospital
Nippon Medical School Tama Nagayama Hospital
Nishi Kobe Medical Center
Nissan Tamagawa Hospital
Nozaki Tokushukai Hospital
Numazu City Hospital
Obihiro Kousei Hospital
Ogaki Municipal Hospital
Ohta Hospital
Ohta Nishinouchi Hospital
Oita Red Cross Hospital
Oita University Hospital
Okayama Red Cross General Hospital
Okayama Saiseikai General Hospital
Okayama University Hospital
Okitama Public General Hospital
Onomichi Municipal Hospital
Osaka City General Hospital
Osaka City University Hospital
Osaka Ekisaikai Hospital
Osaka General Medical Center
Osaka International Cancer Institute
Osaka Medical College Hospital
Osaka Police Hospital
Osaka Red Cross Hospital
Osaki City Hospital
Otsu City Hospital
Rinku General Medical Center
Saga Prefectural Hospital Koseikan
Saga University Hospital
Sagamihara National Hospital
Saiseikai Fukuoka General Hospital
Saiseikai Karatsu Hospital
Saiseikai Noe Hospital
Saiseikai Utsunomiya Hospital
Saiseikai Yokohama Tobu Hospital
Saitama Cancer Center
Saitama Medical University International Medical Center
Saitama Medical University Saitama Medical Center
Sakai City Medical Center
Saku Central Hospital
Sapporo Medical University Hospital
Seikei-kai Chiba Medical Center
Sendai City Hospital
Shiga General Hospital
Shiga University of Medical Science Hospital
Shimane University Hospital
Shin Takeo Hospital
Shinko Hospital
Shinshu University Hospital
Shizuoka Cancer Center
Shizuoka City Shizuoka Hospital
Shizuoka General Hospital
Shizuoka Saiseikai General Hospital
Showa University Hospital
Southern Tohoku General Hospital
St. Luke's International Hospital
St. Marianna University School of Medicine Hospital
St. Mary's Hospital
Steel Memorial Yawata Hospital
Suita Municipal Hospital
Suzuka Chuo General Hospital
Tachikawa Hospital
Takatsuki Red Cross Hospital
Teikyo University Chiba Medical Center
Teikyo University Hospital
Teine Keijinkai Hospital
Tenri Hospital
The Hospital of Hyogo College of Medicine
The Jikei University Daisan Hospital
The Jikei University Hospital
Tochigi Cancer Center
Toho University Ohashi Medical Center
Toho University Omori Medical Center
Toho University Sakura Medical Center
Tohoku University Hospital
Tokai University Hachioji Hospital
Tokai University Hospital
Tokai University Tokyo Hospital
Tokushima Red Cross Hospital
Tokushima University Hospital
Tokyo Dental College Ichikawa General Hospital
Tokyo Medical and Dental University Hospital
Tokyo Medical University Hachioji Medical Center
Tokyo Medical University Hospital
Tokyo Metropolitan Cancer and Infectious Diseases Center Komagome Hospital
Tokyo Metropolitan Tama Medical Center
Tokyo Rosai Hospital
Tokyo University Hospital
Tokyo Women's Medical University Hospital
Tokyo Women's Medical University Medical Center East
Tokyo Women's Medical University Yachiyo Medical Center
Tonan Hospital
Toranomon Hospital
Tosei General Hospital
Toshima Hospital
Tottori Prefectural Central Hospital
Tottori University Hospital
Toyama Prefectural Central Hospital
Toyama University Hospital
Toyonaka Municipal Hospital
Toyota Memorial Hospital
Tsuchiura Kyodo Hospital
Tsukuba University Hospital
Tsuruoka Municipal Shonal Hospital
Tsuyama Chuo Hospital
University Hospital Kyoto Prefectural University of Medicine
University of the Ryukyus Hospital
Wakayama Medical University Hospital
Yamagata Prefectural Central Hospital
Yamagata University Hospital
Yamaguchi University Hospital
Yamanashi Prefectural Central Hospital
Yamanashi University Hospital
Yao Municipal Hospital
Yokohama City Municipal Hospital
Yokohama City University Hospital
Yokohama City University Medical Center
Yokohama Sakae Kyosai Hospital
Yokosuka General Hospital Uwamachi

(Total 334 institutions)

## Patient background

Tables 1, 2, 3, 4, 5, 6, 7, 8

**Table 1:** Age and gender

Age	Male	Female	Cases (%)
≤ 29	12	1	13 (0.2%)
30–39	16	6	22 (0.3%)
40–49	164	59	223 (2.8%)
50–59	917	174	1091 (13.6%)
60–69	2675	431	3106 (38.7%)
70–79	2403	437	2840 (35.4%)
80–89	570	133	703 (8.8%)
90 ≤	10	11	21 (0.3%)
Total	6767	1252	8019

**Table 2:** Performed treatment

Treatments	Cases (%)
Surgery	5038 (62.8%)
Esophagectomy	4910 (61.2%)
Palliative surgery	128 (1.6%)
Chemotherapy and/or Radiotherapy	4062 (50.7%)
Endoscopic treatment	1421 (17.7%)

**Table 3:** Tumor location

Location of tumor	Endoscopic treatment	Surgery		Chemotherapy and/or radiotherapy (%)	Total (%)
	(%)	Esophagectomy (%)	Palliative surgery (%)		
Cervical	48 (3.4%)	163 (3.3%)	10 (7.8%)	256 (6.3%)	384 (4.8%)
Upper thoracic	142 (10.0%)	525 (10.7%)	25 (19.5%)	597 (14.7%)	969 (12.1%)
Middle thoracic	775 (54.5%)	2188 (44.6%)	61 (47.7%)	1864 (45.9%)	3726 (46.5%)
Lower thoracic	369 (26.0%)	1544 (31.4%)	26 (20.3%)	1118 (27.5%)	2264 (28.2%)
EG	61 (4.3%)	356 (7.3%)	5 (3.9%)	165 (4.1%)	470 (5.9%)
E = G	14 (1.0%)	66 (1.3%)		23 (0.6%)	88 (1.1%)
GE	6 (0.4%)	61 (1.2%)		15 (0.4%)	72 (0.9%)
Unknown	6 (0.4%)	7 (0.1%)	1 (0.8%)	45 (0.6%)	46 (0.6%)
Total	1421	4910	128	4062	8019

*E* esophageal, *G* gastric

**Table 4:** Histologic type of biopsy specimens

Histologic types	Endoscopic treatment	Surgery		Chemotherapy and/or radiotherapy (%)	Total (%)
	(%)	Esophagectomy (%)	Palliative surgery (%)		
Squamous cell carcinoma	1101 (77.5%)	4291 (87.4%)	116 (90.6%)	3733 (91.9%)	6911 (86.2%)
Squamous cell carcinoma	867 (61.0%)	2442 (49.7%)	74 (57.8%)	2330 (57.4%)	4377 (54.6%)
Well differentiated	100 (7.0%)	387 (7.9%)	9 (7.0%)	259 (6.4%)	565 (7.0%)
Moderately differentiated	117 (8.2%)	1093 (22.3%)	24 (18.8%)	805 (19.8%)	1448 (18.1%)
Poorly differentiated	17 (1.2%)	369 (7.5%)	9 (7.0%)	339 (8.3%)	521 (6.5%)
Adenocarcinoma	38 (2.7%)	340 (6.9%)	4 (3.1%)	133 (3.3%)	419 (5.2%)
Barrett's carcinoma	33 (2.3%)	91 (1.9%)	1 (0.8%)	25 (0.6%)	133 (1.7%)
Adenosquamous carcinoma	2 (0.1%)	11 (0.2%)		4 (0.1%)	14 (0.2%)
Mucoepidermoid carcinoma		1 (0.0%)			1 (0.0%)
Basaloid carcinoma	2 (0.1%)	31 (0.6%)		16 (0.4%)	39 (0.5%)
Neuroendocrine tumor		1 (0.0%)		1 (0.0%)	2 (0.0%)
Neuroendocrine carcinoma	4 (0.3%)	21 (0.4%)		27 (0.7%)	34 (0.4%)
Undifferentiated carcinoma		6 (0.1%)		4 (0.1%)	9 (0.1%)
Malignant melanoma		15 (0.3%)		6 (0.1%)	19 (0.2%)
Carcinosarcoma		16 (0.3%)	1 (0.8%)	8 (0.2%)	19 (0.2%)
GIST		1 (0.0%)			1 (0.0%)
Adenoid cystic carcinoma		1 (0.0%)			1 (0.0%)
Nonepithelial tumors	2 (0.1%)				3 (0.0%)
Other epithelial tumors	17 (1.2%)	4 (0.1%)		8 (0.2%)	27 (0.3%)
Other tumors	51 (3.6%)	16 (0.3%)		13 (0.3%)	79 (1.0%)
Unknown	171 (12.0%)	64 (1.3%)	6 (4.7%)	84 (2.1%)	308 (3.8%)
Total	1421	4910	128	4062	8019

**Table 5:** Depth of tumor invasion, cT (UICC TNM 7th)

Clinical T	Endoscopic treatment	Surgery		Chemotherapy and/or radiotherapy (%)	Total (%)
	(%)	Esophagectomy (%)	Palliative surgery (%)		
cTX	12 (0.8%)	11 (0.2%)	1 (0.8%)	34 (0.8%)	80 (1.0%)
cT0	6 (0.6%)	4 (0.1%)		3 (0.1%)	14 (0.2%)
cT1a	1139 (80.2%)	247 (5.0%)		102 (2.5%)	1426 (17.8%)
cT1b	196 (13.8%)	1319 (26.9%)	9 (7.0%)	515 (12.7%)	1658 (20.7%)
cT2	4 (0.3%)	832 (16.9%)	4 (3.1%)	609 (15.0%)	1006 (12.5%)
cT3	41 (2.9%)	2223 (45.3%)	49 (38.3%)	2036 (50.1%)	2895 (36.1%)
cT4a	4 (0.3%)	133 (2.7%)	17 (13.3%)	257 (6.3%)	341 (4.3%)
cT4b	19 (1.3%)	141 (2.9%)	48 (37.5%)	506 (12.5%)	599 (7.5%)
Total	1421	4910	128	4062	8019

**Table 6:** Lymph node metastasis, cN (UICC TNM 7th)

Clinical N	Endoscopic treatment	Surgery		Chemotherapy and/or radiotherapy (%)	Total (%)
	(%)	Esophagectomy (%)	Palliative surgery (%)		
cN0	1351 (95.1%)	2278 (46.4%)	31 (24.2%)	1117 (27.5%)	4047 (50.5%)
cN1	38 (2.7%)	1704 (34.7%)	37 (28.9%)	1663 (40.9%)	2318 (28.9%)
cN2	22 (1.5%)	800 (16.3%)	43 (33.6%)	1009 (24.8%)	1301 (16.2%)
cN3	10 (0.7%)	128 (2.6%)	17 (13.3%)	273 (6.7%)	353 (4.4%)
Total	1421	4910	128	4062	8019

**Table 7:** Distant metastasis, cM (UICC TNM 7th)

Clinical M	Endoscopic treatment	Surgery		Chemotherapy and/or radiotherapy (%)	Total (%)
	(%)	Esophagectomy (%)	Palliative surgery (%)		
cM0	1406 (98.9%)	4753 (96.8%)	103 (80.5%)	3513 (86.5%)	7350 (91.7%)
cM1	15 (1.1%)	157 (3.2%)	25 (19.5%)	549 (13.5%)	669 (8.3%)
Total	1421	4910	128	4062	8019

**Table 8:** Clinical Stage (UICC TNM 7th)

Clinical stage	Endoscopic treatment	Surgery		Chemotherapy and/or radiotherapy (%)	Total (%)
	(%)	Esophagectomy (%)	Palliative surgery (%)		
Stage IA	1317 (92.7%)	1268 (25.8%)	7 (5.5%)	388 (9.6%)	2712 (33.8%)
Stage IB	3 (0.2%)	417 (8.5%)	3 (2.3%)	252 (6.2%)	492 (6.1%)
Stage IIA	8 (0.6%)	523 (10.7%)	10 (7.8%)	356 (8.8%)	629 (7.8%)
Stage IIB	15 (1.1%)	522 (10.6%)		389 (9.6%)	609 (7.6%)
Stage IIIA	19 (1.3%)	1169 (23.8%)	14 (10.9%)	952 (23.4%)	1375 (17.1%)
Stage IIIB	9 (0.6%)	514 (10.5%)	14 (10.9%)	488 (12.0%)	642 (8.0%)
Stage IIIC	17 (1.2%)	325 (6.6%)	55 (43.0%)	667 (16.4%)	821 (10.2%)
Stage IV	15 (1.1%)	157 (3.2%)	25 (19.5%)	549 (13.5%)	669 (8.3%)
Unknown	18 (1.3%)	15 (0.3%)		21 (0.5%)	70 (0.9%)
Total	1421	4910	128	4062	8019

## II. Results of endoscopically treated patients in 2013

Tables 9, 10, 11, and Figs. 1, 2, 3.

**Table 9:** Details of endoscopic treatment for curative intent

Treatment details	Cases (%)
EMR	108 (8.0%)
EMR + YAG laser	1 (0.1%)
EMR + MCT/RFA	
ESD	1224 (90.2%)
ESD + EMR	4 (0.3%)
ESD + PDT	
ESD + YAG laser	5 (0.4%)
PDT	2 (0.1%)
YAG laser	13 (1.0%)
Total	1357

*EMR* endoscopic mucosal resection, *PDT* photodynamic therapy, *YAG* yttrium aluminum garnet, *MCT* microwave coagulation therapy, *ESD* endoscopic submucosal dissection

**Table 10:** Complications of EMR/ESD

Complications of EMR/ESD	Cases (%)
None	1298 (92.7%)
Perforation	10 (0.7%)
Bleeding	1 (0.1%)
Mediastinitis	1 (0.1%)
Stenosis	23 (1.7%)
Others	
Unknown	2 (0.1%)
Total	1335

**Table 11:** Pathologic depth of tumor invasion of MER/ESD specimens

Pathological depth of tumor invasion (pT)	Cases (%)
pTX	22 (1.6%)
pT0	7 (0.5%)
pT1a	1111 (82.8%)
pT1b	201 (15.0%)
pT2	
pT3	1 (0.1%)
Total	1342

**Fig. 1:**
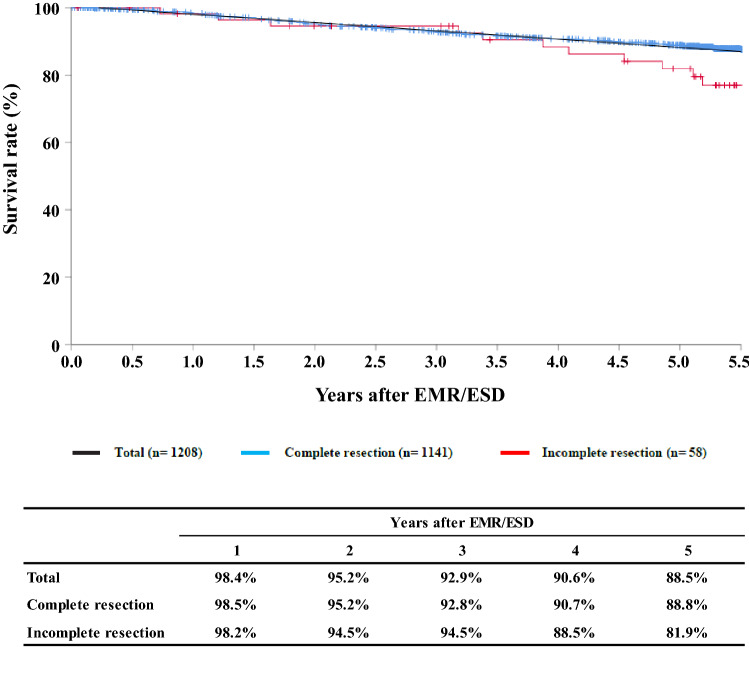
Survival of patients treated with EMR/ESD

**Fig. 2:**
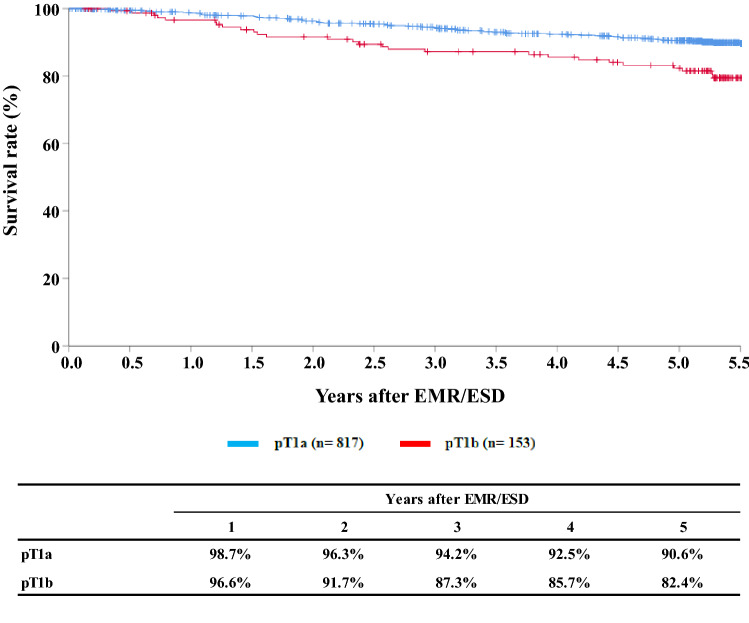
Survival of patients treated with EM/ESD according to the pathological depth of tumor invasion, pT(JES 10th)

**Fig. 3:**
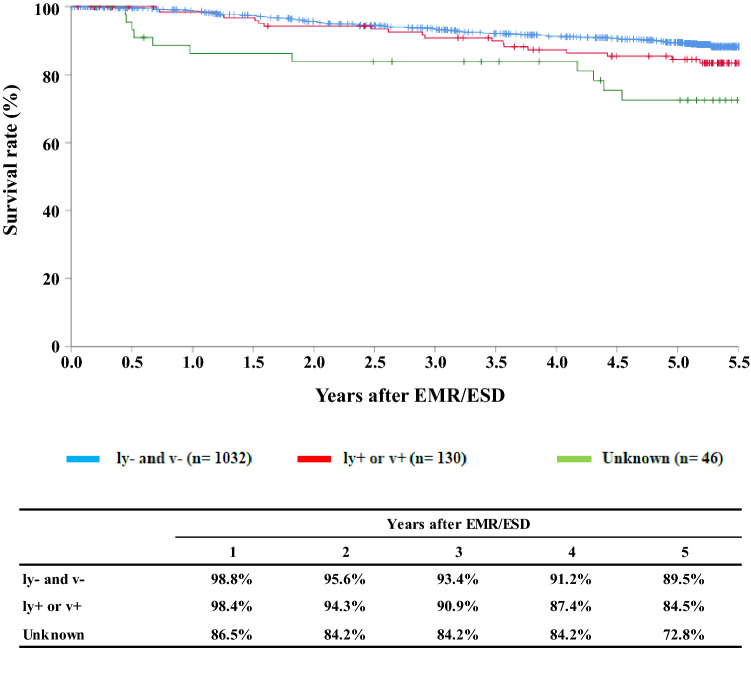
Survival of patients treated with EMR/ESD according to the lymphatic and venous invasion

### III. Results in patients treated with chemotherapy and/or radiotherapy in 2013

Tables 12, 13 and Figs. 4, 5, 6.

**Table 12:** Dose of irradiation (non-surgically treated cases)

Dose of irradiation (Gy)	Definitive		Palliative (%)	Recurrence (%)	Others (%)	Total (%)
	Radiation alone (%)	With chemotherapy (%)				
–29	2 (1.5%)	20 (2.4%)	23 (11.7%)	2 (5.9%)	1 (10.0%)	48 (4.0%)
30–39	4 (3.0%)	16 (1.9%)	33 (16.8%)	1 (2.9%)	2 (20.0%)	56 (4.7%)
40–49	7 (5.3%)	33 (4.0%)	40 (20.4%)	1 (2.9%)	3 (30.0%)	84 (7.0%)
50–59	15 (11.4%)	233 (28.2%)	35 (17.9%)	10 (29.4%)	2 (20.0%)	295 (24.6%)
60–69	98 (74.2%)	505 (61.1%)	63 (32.1%)	19 (55.9%)	2 (20.0%)	687 (57.3%)
70–	6 (4.5%)	18 (2.2%)	1 (0.5%)	1 (2.9%)		26 (2.2%)
Unknown		2 (0.2%)	1 (0.5%)			3 (0.3%)
Total	132	827	196	34	10	1199
Median (min–max)	60.0 (5.4–80.0)	60.0 (2.0–99.0)	50.0 (2.0–70.0)	60.0 (11.0–70.0)	43.2 (26.0–66.0)	60.0 (2.0–99.0)

**Table 13:** Dose of irradiation (surgically treated cases)

Dose of irradiation (Gy)	Preoperative irradiation (%)	Postoperative irradiation (%)
–29	4 (1.4%)	6 (10.0%)
30–39	62 (22.2%)	3 (5.0%)
40–49	177 (63.4%)	5 (8.3%)
50–59	19 (6.8%)	19 (31.7%)
60–69	10 (3.6%)	24 (40.0%)
70–	4 (1.4%)	2 (3.3%)
Unknown	3 (1.1%)	1 (1.7%)
Total	279	60
Median (min–max)	40.0 (2.0–99.0)	55.0 (16.0–75.9)

**Fig. 4:**
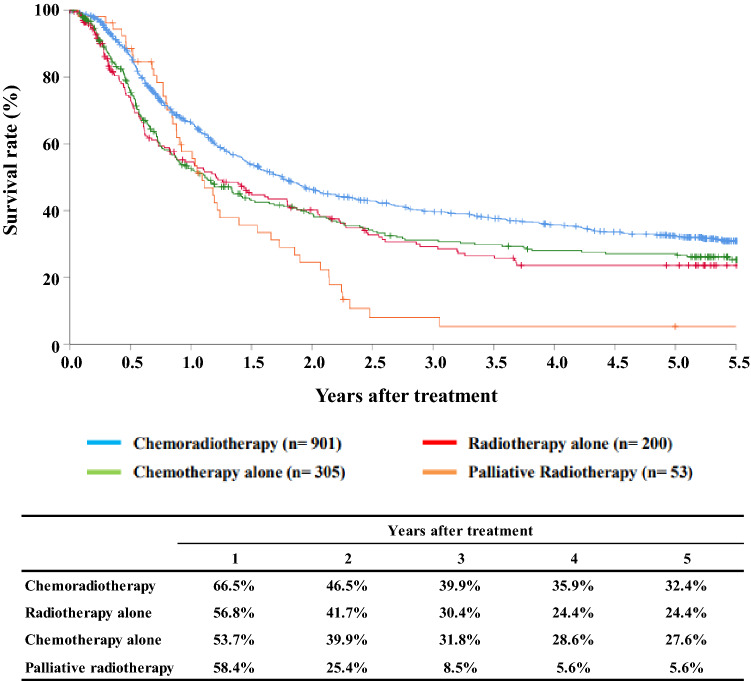
Survival of patients treated with chemotherapy and/or radiotherapy

**Fig. 5:**
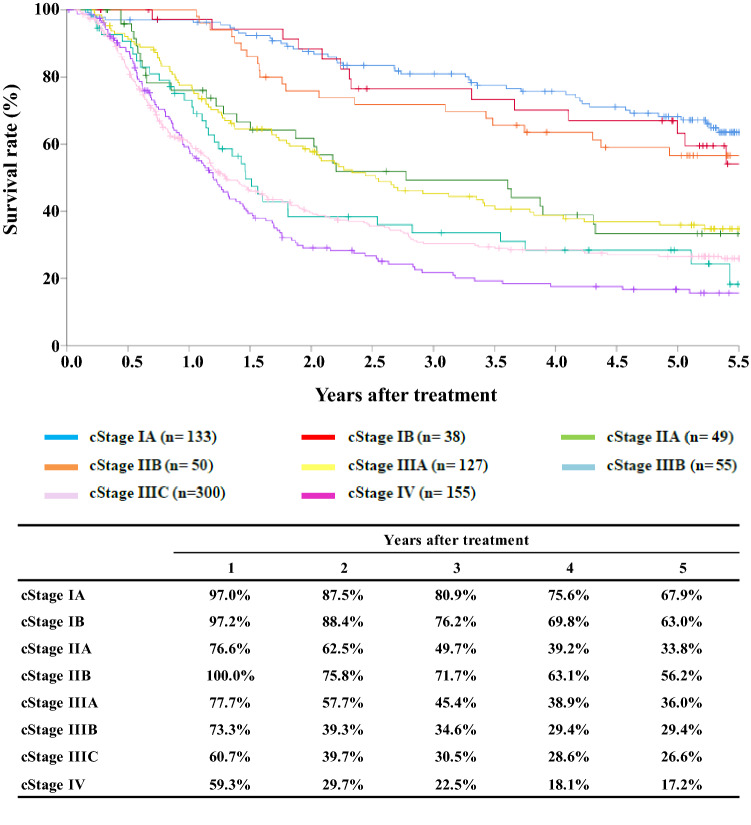
Survival of patients treated with definitive chemoradiotheraphy according to clinical stage (UICC TNM 7th)

**Fig. 6:**
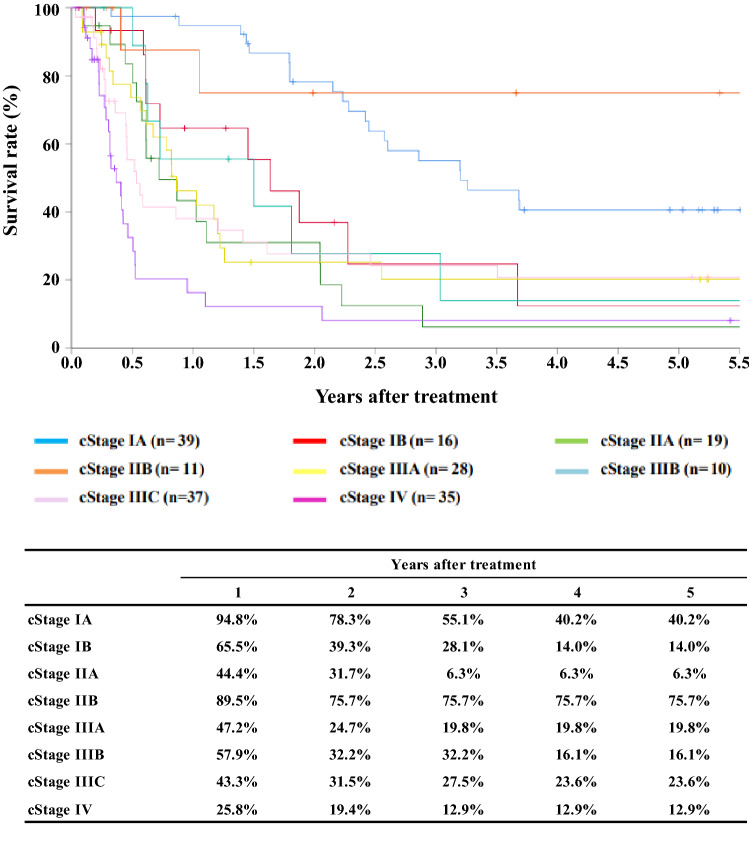
Survival of patients underwent radiotherapy alone according to clinical stage (UICC TNM 7th)

### IV. Results in patients who underwent esophagectomy in 2013

Tables 14, 15, 16, 17, 18, 19, 20, 21, 22, 23, 24, 25, 26, 27, and Figs. 7, 8, 9, 10, 11, 12, 13, 14, 15

**Table 14:** Treatment modalities of esophagectomy

Treatment modalities	Cases (%)
Esophagectomy alone	2336 (47.6%)
Esophagectomy + postoperative chemotherapy	385 (7.8%)
Esophagectomy + postoperative chemoradiotherapy	109 (2.2%)
Esophagectomy + postoperative radiotherapy	34 (0.7%)
Preoperative chemotherapy + esophagectomy	1558 (31.7%)
Preoperative chemoradiotherapy + esophagectomy	286 (5.8%)
Definitive radiotherapy + esophagectomy	6 (0.1%)
Definitive chemoradiotherapy + esophagectomy	101 (2.1%)
Others	95 (1.9%)
Total	4910

**Table 15:** Tumor location

Locations	Cases (%)
Cervical	166 (3.4%)
Upper thoracic	536 (10.9%)
Middle thoracic	2165 (44.1%)
Lower thoracic	1507 (30.7%)
EG	368 (7.5%)
E = G	85 (1.7%)
GE	72 (1.5%)
Unknown	11 (0.2%)
Total	4910

**Table 16:** Approaches to tumor resection

Approaches	Cases (%)
Cervical	135 (2.7%)
Right thoracic	4171 (84.9%)
Left thoracic	63 (1.3%)
Left thoracoabdominal	115 (2.3%)
Abdominal	171 (3.5%)
Transhiatal lower esophagectomy	94 (1.9%)
Transhiatal thoracic esophagectomy	100 (2.0%)
Sternotomy	2 (0.0%)
Others	46 (0.9%)
Unknown	13 (0.3%)
Total	4910

Thoracic includes thoracotomy and thoracoscopic

Abdominal includes laparotomy and laparoscopic

**Table 17:** Video-assisted surgery

Video-assisted surgery	Cases (%)
None	2444 (49.8%)
Thoracoscopy	1072 (21.8%)
Thoracoscopy + laparoscopy	1037 (21.1%)
Thoracoscopy + laparoscopy + mediastinoscopy	5 (0.1%)
Thoracoscopy + laparoscopy + other	
Thoracoscopy + mediastinoscopy	
Thoracoscopy + other	1 (0.0%)
Laparoscopy	237 (4.8%)
Laparoscopy + mediastinoscopy	11 (0.2%)
Laparoscopy + mediastinoscopy + other	11 (0.3%)
Mediastinoscopy	57 (1.2%)
Laparoscopy + other	2 (0.0%)
Others	30 (0.6%)
Unknown	3 (0.1%)
Total	4910

**Table 18:** Fields of lymph node dissection according to the location of tumor

Field of lymphadenectomy	Cervical	Upper thoracic	Middle thoracic	Lower thoracic	Abdominal	E = G	GE	Unknown	Total
None	5 (3.0%)	15 (2.8%)	24 (1.1%)	31 (2.1%)	6 (1.6%)	2 (2.4%)	2 (2.8%)	3 (27.3%)	88 (1.8%)
C	36 (21.7%)	8 (1.5%)	16 (0.7%)	10 (0.7%)					70 (1.4%)
C + UM	21 (12.7%)	1 (0.2%)	1 (0.0%)	4 (0.3%)				1 (9.1%)	28 (0.6%)
C + UM + MLM	10 (6.0%)	14 (2.6%)	47 (2.2%)	15 (1.0%)	1 (0.3%)				87 (1.8%)
C + UM + MLM + A	69 (41.6%)	336 (62.7%)	1098 (50.7%)	532 (35.3%)	62 (16.8%)	10 (11.8%)	1 (1.4%)	5 (45.5%)	2113 (43.0%)
C + UM + A	5 (3.0%)	4 (0.7%)	17 (0.8%)	7 (0.5%)	1 (0.3%)		1		35 (0.7%)
C + MLM				1 (0.1%)					1 (0.0%)
C + MLM + A	3 (1.8%)	6 (1.1%)	14 (0.6%)	9 (0.6%)	1 (0.3%)				33 (0.7%)
C + A	3 (1.8%)	3 (0.6%)	4 (0.2%)	6 (0.4%)			1 (1.4%)		17 (0.3%)
UM	1 (0.6%)	2 (0.4%)	9 (0.4%)	1 (0.1%)	1 (0.3%)				14 (0.3%)
UM + MLM	3 (1.8%)	8 (1.5%)	41 (1.9%)	24 (1.6%)	5 (1.4%)	1 (1.2%)			82 (1.7%)
UM + MLM + A	3 (1.8%)	124 (23.1%)	792 (36.6%)	668 (44.3%)	116 (31.5%)	16 (18.8%)	10 (13.9%)		1729 (35.2%)
UM + A		2 (0.4%)	12 (0.6%	9 (0.6%)	2 (0.5%)				25 (0.5%)
MLM		3 (0.6%)	8 (0.4%)	8 (0.5%)	4 (1.1%)	1 1.2%)	2 (2.8%)		26 (0.5%)
MLM + A	3 (1.8%)	4 (0.7%)	62 (2.9%)	154 (10.2%)	141 (38.3%)	43 (50.6%)	39 (54.2%)		446 (9.1%)
A	4 (2.4%)	6 (1.1%)	20 (0.9%)	28 (1.9%)	28 (7.6%)	12 (14.1%)	16 (22.0%)	2 (18.2%)	116 (2.4%)
Total	166	536	2165	1507	368	85	72	11	4910

*C* bilateral cervical nodes, *UM* upper mediastinal nodes, *MLM* middle-lower mediastinal nodes, *A* abdominal nodes

**Table 19:** Reconstruction route

Route	Cases (%)
None	62 (1.3%)
Subcutaneous	353 (7.2%)
Retrosternal	1971 (40.1%)
Posterior mediastinal	1972 (40.2%)
Intrathoracic	462 (9.4%)
Cervical	49 (1.0%)
Others	26 (0.5%)
Unknown	15 (0.3%)
Total	4910

**Table 20:** Organs used for reconstruction

Organs	Cases (%)
None	88 (1.3%)
Whole stomach	215 (4.3%)
Gastric tube	4114 (83.1%)
Jejunum	249 (5.0%)
Free jejunum	85 (1.7%)
Colon	162 (3.3%)
Free colon	8 (0.2%)
Others	32 (0.6%)
Total organs	4953
Total cases	4822

**Table 21:** Histological classification

Histological classification	Cases (%)
Squamous cell carcinoma	4086 (83.2%)
Squamous cell carcinoma	756 (15.4%)
Well differentiated	750 (15.3%)
Moderately differentiated	1989 (40.5%)
Poorly differentiated	591 (12.0%)
Adenocarcinoma	306 (6.2%)
Barrett's carcinoma	118 (2.4%)
Adenosquamous carcinoma	22 (0.4%)
Mucoepidermoid carcinoma	1 (0.0%)
Basaloid carcinoma	86 (1.8%)
Neuroendocrine tumor	1 (0.0%)
Neuroendocrine carcinoma	32 (0.7%)
Undifferentiated carcinoma	8 (0.2%)
Malignant melanoma	16 (0.3%)
Carcinosarcoma	43 (0.9%)
GIST	2 (0.0%)
Adenoid cystic carcinoma	3 (0.1%)
Sarcoma	2 (0.0%)
Other carcinomas	3 (0.1%)
Other tumors	41 (0.8%)
Unknown	140 (2.9%)
Total	4910

**Table 22:** Pathological depthe of tumor invasion, pT (JES 10th)

Pathological depth of tumor invasion	Cases (%)
pTx	72 (1.5%)
pT0	161 (3.3%)
pT1a	589 (12.0%)
pT1b	1339 (27.3%)
pT2	607 (12.4%)
pT3	1898 (38.7%)
pT4a	138 (0.8%)
pT4b	106 (2.2%)
Total	4910

**Table 23:** Pathological grading of lymph node metastasis, pN (JES 10th)

Lymph node metastasis	Cases (%)
pN0	2335 (47.6%)
pN1	936 (19.1%)
pN2	1000 (20.4%)
pN3	354 (7.2%)
pN4	254 (5.2%)
Unknown	31 (0.6%)
Total	4910

**Table 24:** Pathological grading of lymph node metastasis, pN (UICC TNM 7th)

Lymph node metastasis	Cases (%)
pN0	2361 (48.1%)
pN1 (1–2)	1374 (28.0%)
pN2 (3–6)	757 (15.4%)
pN3 (7–)	366 (7.5%)
Unknown	52 (1.1%)
Total	4910

**Table 25:** Pathological findings of distant organ metastasis, pM (JES 10th)

Distant metastasis (M)	Cases (%)
MX	125 (2.5%)
M0	4715 (96.0%)
M1	70 (1.4%)
Total	4910

**Table 26:** Residual tumor

Residual tumor (R)	Cases (%)
RX	126 (2.6%)
R0	4359 (88.8%)
R1	239 (4.9%)
R2	186 (3.8%)
Total	4910

**Table 27:** Cause of death

Cause of death	Cases (%)
Death due to recurrence	1584 (63.8%)
Death due to other cancer	193 (7.8%)
Death due to other disease (with recurrence)	50 (2.0%)
Death due to other disease (without recurrence)	330 (13.3%)
Death due to other disease (recurrence unknown)	15 (0.6%)
Operative death^a^	38 (1.5%)
Postoperative hospital death^b^	59 (2.4%)
Unknown	213 (8.6%)
Total of death cases	2482

^a^Operative death means death within 30 days after operation in or out of hospital. Operative mortality rate: 0.77%

^b^Hospital death is defined as death during the same hospitalization, regardless of department at time of death. Hospital mortality rate: 1.98%

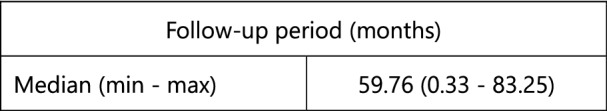

**Fig. 7:**
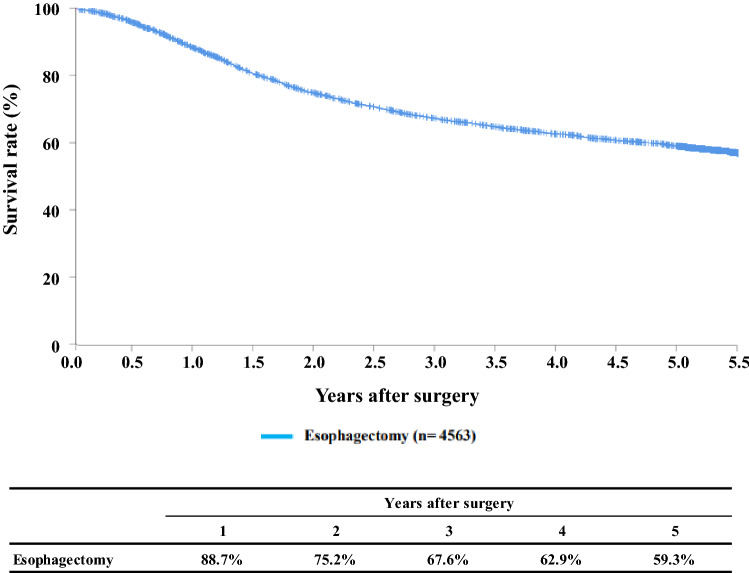
Survival of patients who underwent esophagectomy

**Fig. 8:**
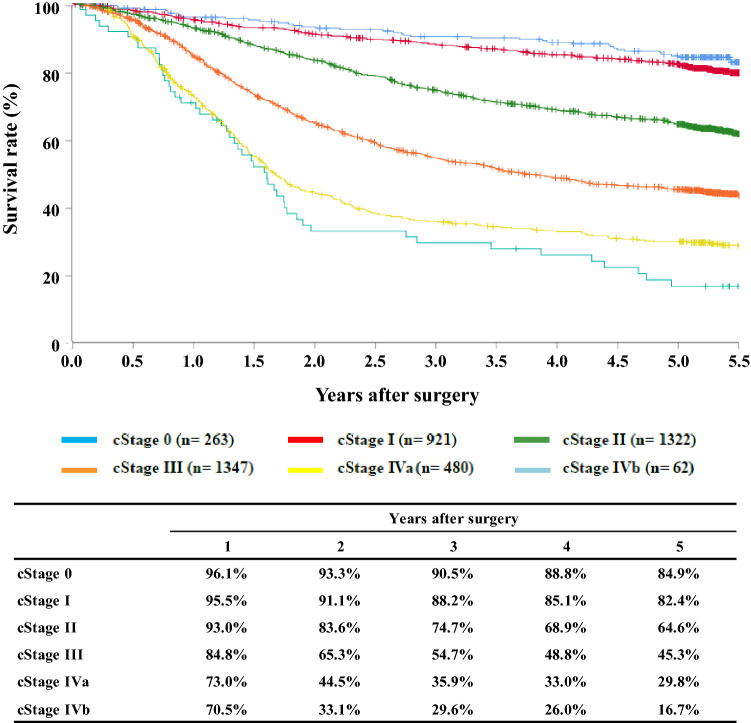
Survival of patients who underwent eseophagectomy according to clinical stage (JES 10th)

**Fig. 9:**
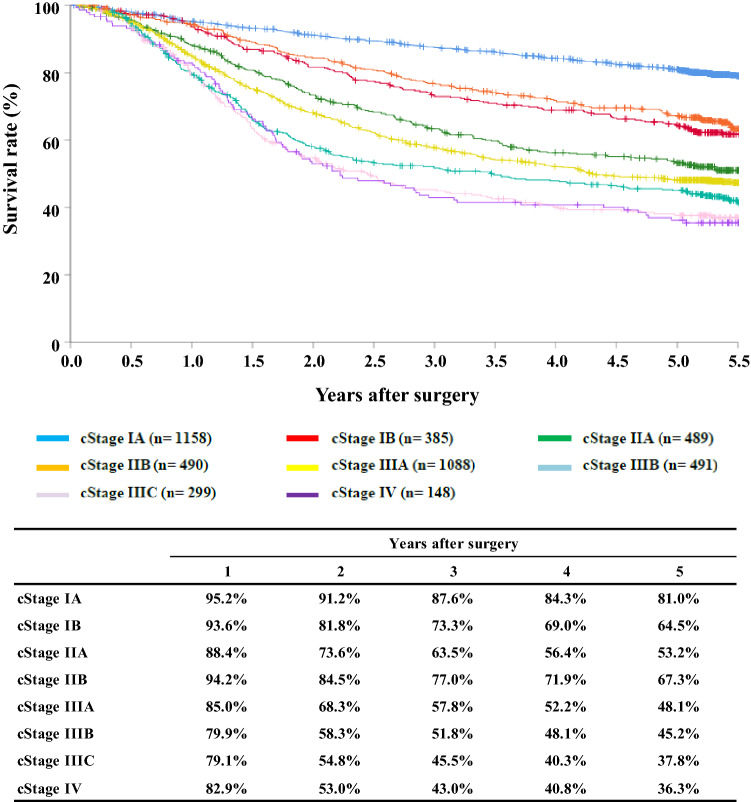
Survival of patients who underwent esophagectomy according to clinical stage (UICC TNM 7th)

**Fig. 10:**
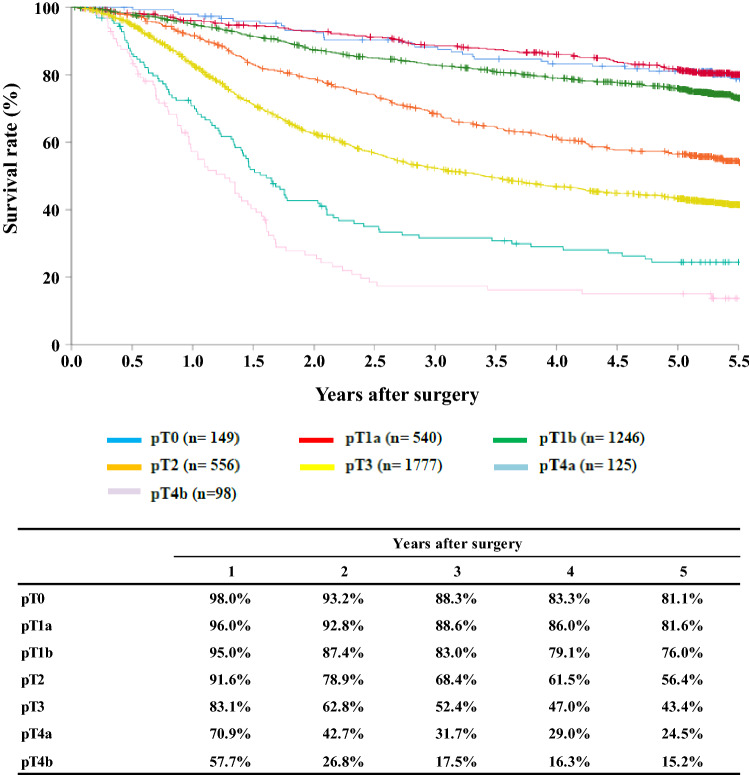
Survival of patients who underwent esophagectomy according to the depth of tumor invasion, pT (JES 10th)

**Fig. 11:**
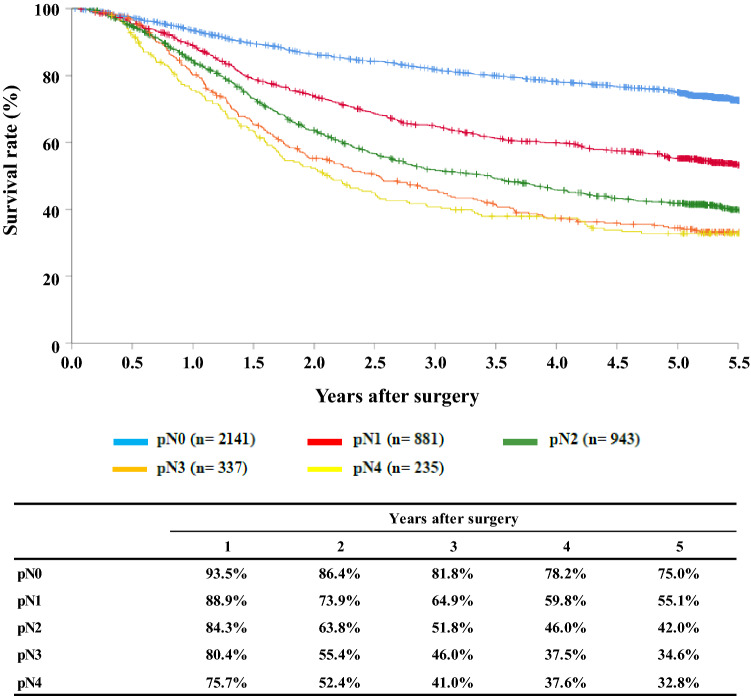
Survival of patients who underwent esophagectomy according to lymph node metastasis (JES 10th)

**Fig. 12:**
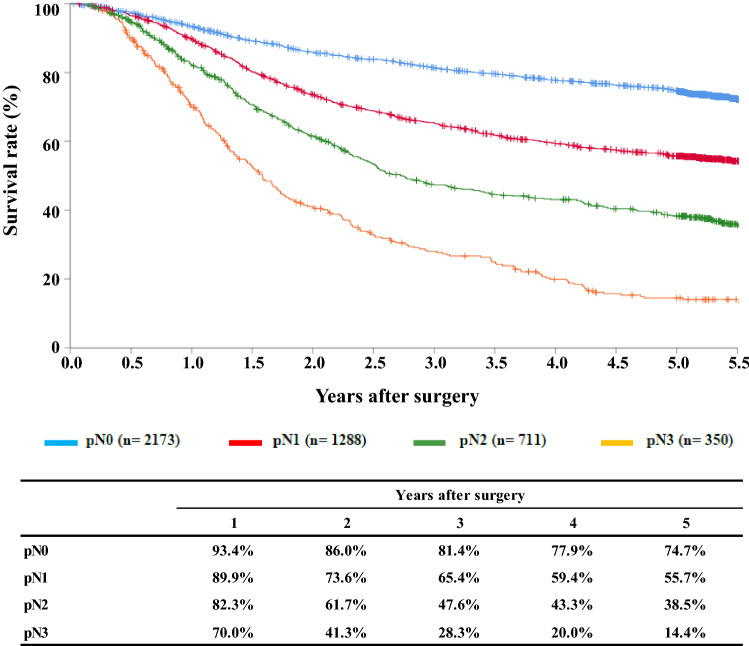
Survival of patients who underwent esophagectomy according to lymph node metastasis (UICC TNM 7th)

**Fig. 13:**
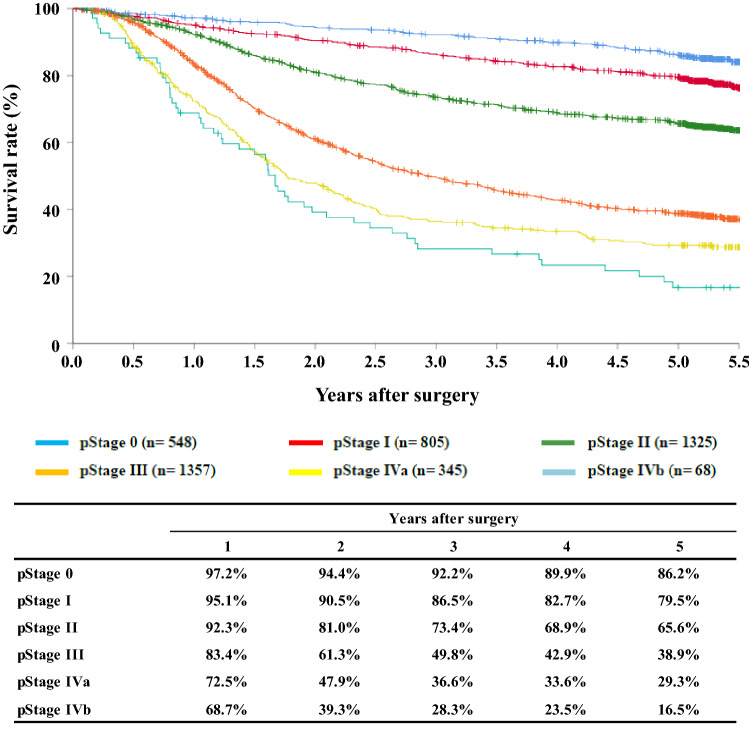
Survival of patients who underwent esophagectomy according to pathological stage (JES 10th)

**Fig. 14:**
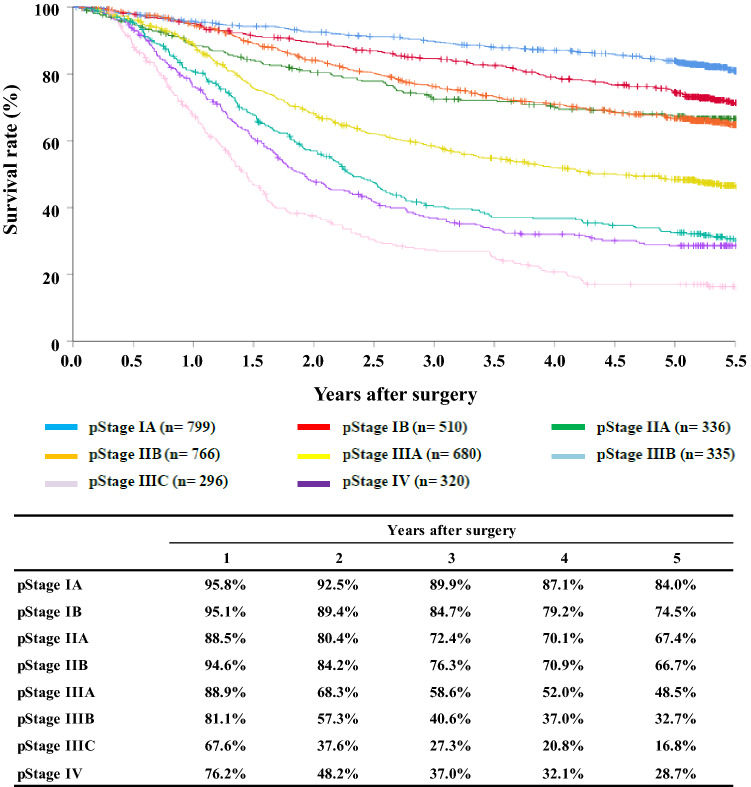
Survival of patients who underwent esophagectomy according to pathological stage (UICC TNM 7th)

**Fig. 15:**
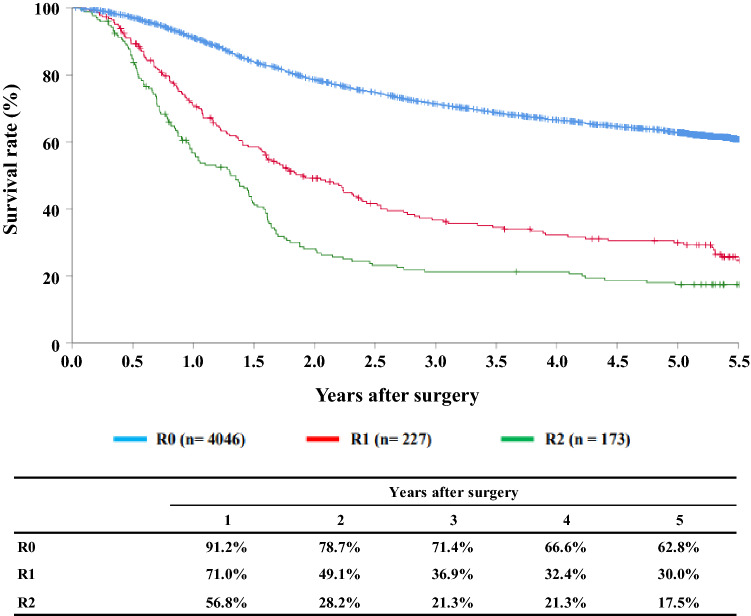
Survival of patients who underwent esophagectomy according to residual tumor (R)
